# Candidate Genes of Regulation of Skeletal Muscle Energy Metabolism in Athletes

**DOI:** 10.3390/genes12111682

**Published:** 2021-10-23

**Authors:** Olga V. Balberova, Evgeny V. Bykov, German V. Medvedev, Margarita A. Zhogina, Kirill V. Petrov, Marina M. Petrova, Mustafa Al-Zamil, Vera V. Trefilova, Polina S. Goncharova, Natalia A. Shnayder

**Affiliations:** 1 The Research Institute of Olympic Sports, Ural State University of Physical Culture, 454091 Chelyabinsk, Russia; bev58@yandex.ru; 2 Department of Hand Surgery with Microsurgical Equipment, Vreden National Medical Research Center of Traumatology and Orthopedics, 195427 St. Petersburg, Russia; dr.medvedev.g@yandex.ru (G.V.M.); zhoginamargo@mail.ru (M.A.Z.); 3 Department of Physical and Rehabilitation Medicine with a Postgraduate Course, Professor V.F. Voino-Yasenetsky Krasnoyarsk State Medical University, 660022 Krasnoyarsk, Russia; kllpetrov@mail.ru (K.V.P.); stk99@yandex.ru (M.M.P.); 4 Department of Physiotherapy, Peoples’ Friendship University of Russia, 117198 Moscow, Russia; alzamil@mail.ru; 5 The Neurological Department No. 16, The Hospital for War Veterans, 193079 St. Petersburg, Russia; vera.v.trefilova@yandex.ru; 6 Center of Personalized Psychiatry and Neurology, V.M. Bekhterev National Medical Research Center for Psychiatry Neurology, 192019 St. Petersburg, Russia; po.gon4arova@yandex.ru; 7 Shared Core Facilities Molecular and Cell Technologies, V.F. Voino-Yasenetsky Krasnoyarsk State Medical University, 660022 Krasnoyarsk, Russia

**Keywords:** personalized medicine, sports genetics, candidate genes, single nucleotide variant, polymorphism, energy metabolism, skeletal muscles, athlete

## Abstract

All biological processes associated with high sports performance, including energy metabolism, are influenced by genetics. DNA sequence variations in such genes, single nucleotide variants (SNVs), could confer genetic advantages that can be exploited to achieve optimal athletic performance. Ignorance of these features can create genetic “barriers” that prevent professional athletes from pursuing a career in sports. Predictive Genomic DNA Profiling reveals single nucleotide variations (SNV) that may be associated with better suitability for endurance, strength and speed sports. (1) Background: To conduct a research on candidate genes associated with regulation of skeletal muscle energy metabolism among athletes. (2) Methods: We have searched for articles in SCOPUS, Web of Science, Google Scholar, Clinical keys, PubMed, e-LIBRARY databases for the period of 2010–2020 using keywords and keywords combinations; (4) Conclusions: Identification of genetic markers associated with the regulation of energy metabolism in skeletal muscles can help sports physicians and coaches develop personalized strategies for selecting children, teenagers and young adults for endurance, strength and speed sports (such as jogging, middle or long distance runs). However, the multifactorial aspect of sport performances, including impact of genetics, epigenetics, environment (training and etc.), is important for personalized strategies for selecting of athletes. This approach could improve sports performance and reduce the risk of sports injuries to the musculoskeletal system.

## 1. Introduction

**Figure 1:**
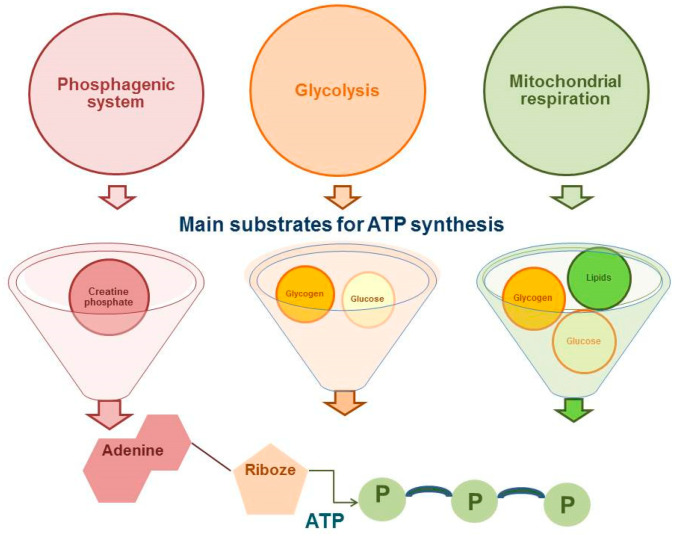
Metabolic pathways of adenosine triphosphate (ATP) synthesis.

The proportion of competitive period in the annual training cycle in cyclic sports (such as running disciplines of athletics) has significantly increased [1]. This makes high demands on the physiological and biochemical aspects of training athletes on the first place. It should be noted that running disciplines vary from sprint, which last for a few seconds, to marathon taking hours to run [2]. Accordingly, the competitive result in cyclic sports will be determined, firstly, by the availability of adenosine triphosphate (ATP) as the main substrate of energy supply and, secondly, by the number of skeletal muscle motor units involved. As presented in Figure 1, the energy supply for muscle activity is provided by three energy systems: phosphogenic pathway, glycolytic pathway, and mitochondrial respiration (Figure 1).

All energy systems for the synthesis of ATP molecules use a different substrate, involve a different number of reactions, resulting in having a different regeneration rate of ATP molecules and different metabolic products, which will determine the contribution of each system to the process of fatigue of the athlete’s skeletal muscles and a movement speed decrease, which is important in cyclic sports [3,4,5].

Creatine phosphate (CRF) and muscle glycogen cleavage provides phosphorylation at the substrate level without the participation of oxygen. This pathway of ATP synthesis is very important in short-distance running (sprint), where the load is characterized by very high intensity. At the same time, ATP reserves are quickly exhausted, as a result, fatigue of the athlete’s skeletal muscles and a decrease in movement speed occur. An important regulator of skeletal muscle energy metabolism during high-intensity physical exertion is AMP deaminase (AMPD), which shifts the myokinase reaction towards ATP production. Thus, ATP resynthesis is maintained during muscle fatigue [6].

With an increase in the running distance, oxidative phosphorylation (the mitochondrial pathway of ATP synthesis) becomes more significant in ATP production, which is realized with the obligatory participation of oxygen due to carbohydrates and lipids. Athletic performance in running in this case will largely depend on the balance between lipid-carbohydrate metabolism, as well as on the oxidative capacity of skeletal muscles. It is from these positions that PPAR transcription factors and their activators are of scientific interest in the field of sports medicine [7].

Another important factor affecting athletic success in running short, medium or long distances is the ratio of muscle fibers in an athlete’s skeletal musculature. Skeletal muscle fibers are usually classified as type I (oxidative/slow) and type II (glycolytic/fast) fibers. Type I fibers are rich in mitochondria (mitochondrial respiration is carried out in them) mainly use oxidative metabolism for energy production. It is this type of fiber that is associated with the high capabilities of an athlete in endurance sports (long-distance running). Type II fibers have a low content of mitochondria and oxidative enzymes and depend, to a greater extent, on glycolytic metabolism as the main source of energy. This type of fiber is associated with the high capabilities of an athlete in speed and power sports (sprint) [8].

Thus, the energy supply of muscular activity and the ratio of different types of fibers in skeletal muscles are important factors determining an athlete’s performance in cyclic sports. Consequently, the identification of genetic markers, single nucleotide variants (SNVs), associated with the metabolic and contractile efficiency of skeletal muscles is a priority area of sports genetics. The identification of an athlete’s predisposition to better tolerance of anaerobic or aerobic loads contributes to the rational choice of sports loads and the prevention of sports injuries [5].

The most frequent genes are used further. Big candidates might be lost because to recent and few publications are yet available (some genes at play in myogenesis and homeostasis have been recently discovered these past few years).

In addition, when selecting children and adolescents for sports, it is important to remember that mutations of some of these genes can cause monogenic hereditary diseases (Table 1). Intensive training process can lead to the development of these diseases at an earlier age and to more severe consequences if these diseases were asymptomatic earlier or they are characterized by a later onset.

Objective of the thematic review—to conduct a research on candidate genes associated with regulation of skeletal muscle energy metabolism in athletes.

## 2. Materials and Methods

The search for full-text articles in the SCOPUS, Web of Science, Google Scholar, Clinical keys, PubMed, e-LIBRARY databases for as for 2010 to 2020 was carried out. We used keywords and their combinations: “personalized medicine”; “sports genetics”; “candidate genes”; “single nucleotide variant”; “polymorphism”; “energy metabolism”; “skeletal muscles”; “athlete”. The results of open observational associative genetic case-control studies, genome-wide studies, Cochrane reviews published in English and Russian were analyzed. Despite an in-depth search, it is possible that some publications could have been missed.

## 3. Results

According to our analysis of studies of candidate genes encoding structural proteins and enzymes involved in the regulation of energy metabolism in skeletal muscles, the researchers’ interest in sports genetics has been increasing in recent years. The most studied are 6 candidate genes (Table 2), the expression level of which differs in skeletal muscles, myocardium, and lungs (Table 3), which is important to consider when translating the results of genetic research into real sports practice.

### 3.1. Gene AMPD1

**Figure 2:**
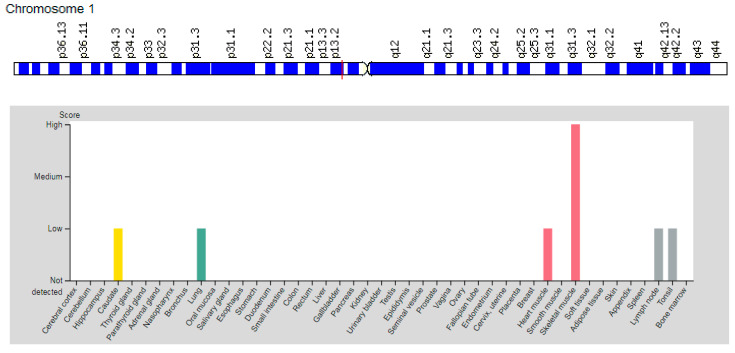
Location of the *AMPD1* gene (**a**) and tissue expression of the adenosine monophosphate deaminase-1, muscle (**b**).

The *AMPD1* gene (Adenosine Monophosphate Deaminase-1 (Muscle)) is located on chromosome 1p13.2 (Figure 2a). The gene catalyzes the deamination of adenosine monophosphate (AMP) to inosine monophosphate (IMP) in skeletal muscle and plays an important role in the purine nucleotide cycle [10]. This enzyme activity is presumed to be important in skeletal muscle because a metabolic myopathy develops in individuals with an inherited deficiency of AMPD1. Expression of the *AMPD1* gene is higher in skeletal muscle compared to the myocardium and lungs (Table 3). Generally, the AMPD1 is expressed predominantly in skeletal muscle (Figure 2b), in which transcript abundance is controlled by stage-specific and fiber type-specific signals.

AMP deaminase (AMPD) is an important regulator of skeletal muscle energy metabolism during exercise. During intense exercise, ATP stores can be depleted. The myokinase mechanism of anaerobic ATP re-synthesis begins to work. This leads to the formation of IMP forms. AMPD shifts the myokinase response towards ATP production. Thus, ATP re-synthesis is supported during muscle fatigue [6].

The AMPD enzyme, which catalyzes the deamination reaction, plays an important role in the purine nucleotide cycle. It has been shown that AMPD stabilizes the energy charge near the actively working myosin ATPase at the ends of the A-disk, mainly in rapidly contracting myofibrils [12].

The skeletal muscle isoform M of the AMPD enzyme is encoded by the *AMPD1* gene. AMPD deficiency in humans can be caused by a single nucleotide substitution of cytosine for thymine (C34T, rs17602729) in exon 2 of the *AMPD1* gene. As a result of this change, the glutamine codon is converted to a stop codon. The protein chain is terminated, and the AMPD enzyme becomes catalytically inert [13]. Individuals with low AMPD enzyme activity cannot effectively perform short-term, high-intensity exercise, which can impair athletic performance.

In the study by Ahmetov et al. [13] a lower frequency of the T allele of the *AMPD1* gene in effective athletes compared to the control group (not involved in sports) was found. The authors concluded that the SNV C34T of the *AMPD1* gene is associated with the tolerance of intense anaerobic exercise in athletes. On the one hand, it is indicated that the C allele can be considered as a biomarker associated with high capabilities to perform speed-strength exercise. On the other hand, the T allele can be considered as a biomarker associated with low tolerance of skeletal muscles to physical activity in anaerobic energy supply mode. Therefore, the CC homozygotes have high AMPD enzyme activity in skeletal muscle, while CT heterozygotes have intermediate enzymatic activity. In contrast the TT homozygotes have very low AMPD enzymatic activity (16% of normal enzyme activity).

The AMPD1 isoform is predominant in all mammalian skeletal muscle fibers. However, the expression of the *AMPD1* gene may contribute to quantitative variations in the total enzyme activity in muscle groups with different fiber composition. However, the highest expression of the *AMPD1* gene was found in fast twitch fibers of skeletal muscle [14].

Ginevičienė et al. [15] have studied the frequency distribution of the alleles/genotypes C34T of the *AMPD1* gene in Lithuanian athletes-runners, divided into three groups: sprint, middle and long distances. The authors compared exercise tolerance among the studied athletes and its association with alleles/genotypes. The control group consisted of randomly selected non-athlete Lithuanian volunteers. The results of the study showed that the frequency of the homozygous TT genotype was 2.4% in the control group, while it was absent in the group of athletes. The highest frequency of the homozygous CC genotype was found in athletes-sprinters (86.3%) compared to endurance oriented marathon athletes (72.9%) and middle-distance runners (67.1%). The frequency of the homozygous CC genotype in the control group was 74.2%. Thus, the SNV C34T of the *AMPD1* gene is not associated with the phenotype of aerobic performance of skeletal muscles in Lithuanians. At the same time, the value of short-term explosive muscle strength (based on the vertical jump test) among athletes in the sprint/power group, was significantly higher in carriers of the homozygous CC genotype than in other groups (*p* < 0.05). The authors concluded that there is a significant association of the homozygous CC genotype with the anaerobic activity of skeletal muscles in athletes-runners.

Rubio [12] researched the effect of the T allele (SNV C34T) of the *AMPD1* gene on physical performance in elite endurance athletes. The study involved 104 elite athletes: cycling and Olympic-class runners (running 1500 m, 5000 m and 10,000 m). The hypothesis was that in elite endurance athletes, the frequency distribution of the minor T allele is lower than in the general population, and the homozygous TT genotype makes it unlikely to achieve elite status. No statistically significant differences were found in the indicators of maximum oxygen consumption (MOC) in carriers of the CT and CC genotypes in the studied groups. In this regard, the authors suggested that in the process of natural selection in achieving the status of elite athletes, a partial metabolic deficit in heterozygous elite athletes (that is a decrease in the turnover of purine nucleotides) is compensated by other training adaptations. An increase in blood flow and oxidative phosphorylation in working muscles can be considered as possible compensatory mechanisms.

Thus the presence of the C allele (SNV C34T) of the *AMPD1* gene can be considered as a biomarker associated with the physical characteristics of sprint and power. This can help in the selection of elite athletes who need effective performance of anaerobic sports activities. The T allele is an unfavorable factor for athletics in sprint/strength sports.

### 3.2. PPARG Gene

**Figure 3:**
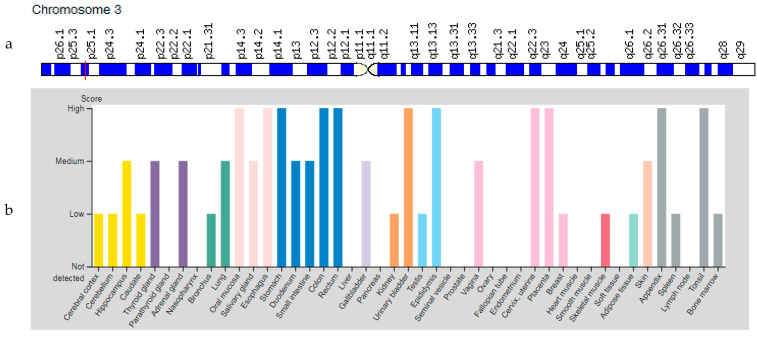
Location of the *PPARG* gene (**a**) and tissue expression of the peroxisome proliferator activated receptor gamma (**b**).

The *PPARG* (Peroxisome Proliferator Activated Receptor gamma) gene is located on chromosome 3p25.2 (Figure 3a). The gene encodes a member of the peroxisome proliferator-activated receptor (PPAR) subfamily of nuclear receptors. Upon activation by a ligand, the nuclear receptor binds to DNA-specific PPAR response elements and modulates the transcription of its target genes, such as acyl-CoA oxidase. Hence, it controls the peroxisome beta-oxidation pathway of fatty acids. This protein is the key regulator of adipocyte differentiation and glucose homeostasis [10]. The *PPARG* gene is expressed in various organs and tissues, including skeletal muscles (Figure 3b).

Due to its role in lipid and carbohydrate metabolism, the *PPARG* gene is often described as a candidate gene associated with obesity and other obese phenotypes. However, it is also currently being considered in the context of its potential role in the functional responses of the human body to exercise [16]. Since the performance of athletes in cyclic sports largely depends on the balance between lipid-carbohydrate metabolism and the precise use of metabolic substrates, PPAR transcription factors and their coactivators are of scientific interest in the field of sports medicine.

*PPARG* is expressed in skeletal muscle, brown adipose tissue, heart and brain, videlicet, in those tissues where increased fat catabolism occurs to obtain a large amount of energy substrates and activate energy supply processes [17]. The main function of the PPARG protein encoded by this gene is the regulation of lipid metabolism, glucose and energy homeostasis, as well as body weight control. In addition, PPARG is a central regulator of adipogenesis and the switch of metabolism from carbohydrate to fat [18].

The most studied SNV of the *PPARG* gene is C34G (rs18012825), which is a substitution of nucleotide C for G at the 34-th position of exon 2, which leads to a substitution of proline for alanine (Pro12Ala) at amino acid position 12 of the PPARG2 protein isoform. The following phenotypes were identified, the different genotypes resulting from this SNV: Pro/Pro-homozygotes for the common (major) C allele; Pro/Ala-rare (minor) G allele heterozygotes; Ala/Ala are homozygotes for the rare (minor) G allele. According to previously obtained data [19,20,21], restoration of tissue sensitivity to insulin is associated with less active lipolysis in adipose tissue and glycolysis in the liver in carriers of the G allele (Ala), which leads to activation of the consumption of free fatty acids by muscle tissue and to reduce their local level.

Kahara et al. [22] identified a high association of the G allele (Ala) with increased sensitivity to insulin, which confirms the conclusion about the enhancement of the anabolic effect of insulin on muscle tissue. Thus, the presence of this allele can give an advantage in speed-strength sports [16,22].

The studies of Ilyutik et al. [23] (2017) investigated the relationship between anthropometric and strength indicators and genotypes associated with this SNV of the *PPARG* gene, which regulates muscle metabolism. Skaters with genotype G/G (Ala/Ala) have a statistically significant tendency to increase the mass of bone and muscle tissue (*p* > 0.005). Skaters—carriers of the homozygous C/C genotype (Pro/Pro) are characterized by large girth sizes, greater thickness of skin and fat folds, and a higher content of the fat component in the total body weight. Carriers of the heterozygous C/G genotype (Pro/Ala) have intermediate values of anthropometric parameters. With regular physical exertion of a speed-strength nature, athletes with a homozygous G/G genotype (Ala/Ala) of the *PPARG* gene increase muscle mass faster due to a decrease in the fat component compared to carriers of other genotypes (C/C; C/G). In the competitive period, skaters with a heterozygous C/G genotype (Pro/Ala) showed a statistically significant higher blood lactate concentration after performing a cycling exercise (10.6 ± 1.03 mmol/L) compared with athletes with a homozygous genotype for the major C allele (Pro/Pro)—6.9 ± 1.54 mmol/L (*p* < 0.05). Results suggest that skaters carriers of the minor G allele (Ala) of the *PPARG* gene display a predisposition to better tolerance of anaerobic loads, and accordingly to the development of speed-strength qualities due to increased glucose utilization during the glycolytic mechanism of energy supply [23].

Mansoori et al. (2015) showed that this SNV of the *PPARG* gene was associated with the level of body mass index (BMI). The presence of the minor G allele (Ala) is associated with an increase in BMI by 0.29 units, and this association was observed in both sexes [24].

Ahmetov et al. [16,25] considered this SNV as a factor influenced by genetics. The authors studied the associations of genotypes of this SNV with aerobic and anaerobic parameters in Russian athletes. The frequency of the major C allele of the *PPARG* gene in elite athletes was higher compared to the control group without achieving statistical significance of differences (90.1% versus 83.6%, respectively), and the frequency of the minor G allele (Ala) was significantly higher in elite athletes compared to the control group (23.1% versus 16.2%, respectively). In addition, hypertrophy of muscle fibers in carriers of the minor G allele (Ala) was observed [26]. The authors concluded that the studied SNV of the *PPARG* gene can be considered as a genetic biomarker that allows predicting high physical performance in speed-strength sports.

Maciejewska-Karlowska et al. [27] conducted a study of the association between genotypes of SNV C34G (rs1801282) of the *PPARG* gene and the sports status among Polish athletes. All participants in this study were divided into four subgroups depending on the specifics of training (endurance, strength, speed among athletes and a control group of non-athletes). The following indicators were studied: the relative contribution of the aerobic/anaerobic system; time of performance of competitive exercises; the load intensity in each sport. The conducted study confirmed a statistically significant higher frequency of the minor G allele (Ala) (compared to the control group) in the subgroup of strength athletes performing short-term and very intense loads, characterized by predominant anaerobic energy production.

Thus, the minor G allele (SNV C34G) of the *PPARG* gene is associated with the status of an elite athlete training in speed-strength sports with a predominantly anaerobic focus of physical activity.

### 3.3. PPARGC1A Gene

**Figure 4:**
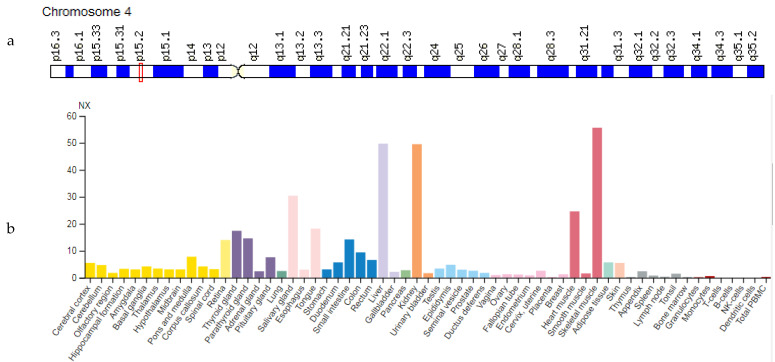
Location of the *PPARGC1A* gene (**a**) and tissue expression of the peroxisome proliferator-activated receptor gamma coactivator 1 Alpha (**b**).

The *PPARGC1A* gene (Peroxisome Proliferator-Activated Receptor gamma Coactivator 1 Alpha) is located on chromosome 4p15.2 (Figure 4a). The protein encoded by this gene is a transcriptional coactivator that regulates genes involved in energy metabolism. It provides a direct link between external physiological stimuli and regulation of mitochondrial biogenesis and is the main factor regulating the determination of muscle fiber type. PPARGC1A can also be involved in blood pressure control, regulation of cellular cholesterol homeostasis, and the development of obesity [10]. Generally, the *PPARGC1A* gene is expressed predominantly in skeletal muscle (Figure 4b).

High expression of *PPARGC1A* is noted in metabolically active tissues with many mitochondria and oxidative phosphorylation, such as heart and skeletal muscle (Table 3).

SNVs are described in the coding region of the *PPARGC1A* gene, which are associated with muscle energy metabolism. SNV C23815662T (rs8192678) leads to the replacement of glycine with serine (Gly482Ser), which has a functional significance in the adaptation of skeletal muscles to physical stress [7,28,29,30]. There is evidence that this SNV is associated with changes in blood lipids and insulin sensitivity. Carriers of Ser have a higher level of low density lipoproteins in the blood serum and higher insulin resistance compared to carriers of Gly, as well as reduced energy metabolism of skeletal muscles [31,32].

A number of studies are devoted to the search for an association between genotypes and alleles C23815662T (rs8192678) of the *PPARGC1A* gene and predisposition to high sports performance both in speed-strength sports and in endurance athletes [7,33].

The ability to engage in prolonged physical activity without significant moving speed reduction (eg, long-distance running) is a process that uses oxidative metabolism [34]. Acute physical activity causes oxidative stress, mobilizes the inflammatory response, enhancing the higher expression of *PPARGC1A*. An increase in the level of *PPARGC1A* mRNA and its overexpression increases the oxidative capacity of skeletal muscles, prevents the development of fatigue in contracting skeletal muscles. As a result, the athlete is able to cope with physical activity for a longer time [7].

The process of switching the type of muscle fibers is important, in particular the transition from glycolytic type IIb to types IIa and I rich in mitochondria, which is associated with high athletic capabilities of the individual in endurance sports. The number of mitochondria in the involved muscle fibers probably determines the power of the load at the level of MOC [8].

*PPARGC1A* has not only been identified as a major regulator of mitochondrial biogenesis, but it has also been shown to regulate proteins involved in angiogenesis and antioxidant protection, as well as affect the expression of inflammatory markers [34,35]. Maciejewska et al. (2012) [34] studied the distribution of the C23815662T (rs8192678) SNV alleles of the *PPARGC1A* gene in a group of Polish athletes. It was found that the minor allele T (Ser) is underrepresented in the cohort of athletes compared to the control group of non-athletes (*p* < 0.0001). A statistically significant low frequency of the T allele (Ser) was observed among Polish athletes training for both long and short distances (*p* = 0.019, *p* = 0.022 respectively). The authors found that the T allele (Ser) is associated with a decrease in aerobic capacity, while the major C allele (Gly), on the contrary, can be considered a biomarker of high aerobic capacity and quality of endurance.

Similar data were obtained by Ahmetov et al. (2009) [36]. The authors examined the association between heterozygous carriage of C/T and the status of marathon athletes, as well as the ratio of slow twitch muscle fibres (types IIa and I) and maximum oxygen consumption. The study involved 1423 Russian athletes and 1132 non-athletes were included in the control group. In conclusion, the authors confirm the significance of the C (Gly) allele of the *PPARGC1A* gene in the elite status of athletes.

Studies have shown that the *PPARGC1A* gene plays an important part in maintaining the expression of mitochondrial metabolic and antioxidant enzymes in skeletal muscles and affects training-induced adaptation of mitochondrial proteins in skeletal muscles. Regular aerobic exercises increase the expression/activity of membrane transporters and mitochondrial metabolic enzymes, and increase capillarization in skeletal muscles, altogether increasing the oxidative capacity of muscle fibres and the ability of myocytes to oxidize carbohydrates and fatty acids. This increases the expression of antioxidant defense enzymes [23], potentially providing better protection against reactive oxygen species in skeletal muscles.

### 3.4. Gene PPARA

**Figure 5:**
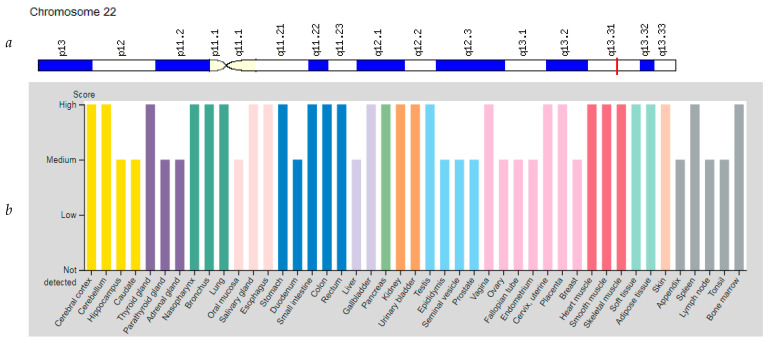
Location of the *PPARA* gene (**a**) and tissue expression of the peroxisome proliferator activated receptor Alpha (**b**).

The *PPARA* (Peroxisome Proliferator Activated Receptor Alpha) gene is located on chromosome 22q13.31 (Figure 5a). The protein encoded by this gene is involved in the regulation of energy metabolism, since it regulates the expression of genes encoding several key muscle enzymes involved in fatty acid oxidation [10,37]. The *PPARA* gene is activated under conditions of energy deprivation, promoting the absorption, utilization and catabolism of fatty acids. The gene is also involved in the immune responses of endurance athletes, as this exercise promotes the activation of the mitochondrial pathway for fatty acid oxidation. The *PPARA* gene is expressed at a high level in tissues that catabolize fatty acids, such as liver, skeletal muscle, myocardium (Figure 5b). The expression level of *PPARA* is higher in type I (slow twitch) muscle fibres than in type II (fast twitch) muscle fibres [38].

The most frequently analyzed SNV in this gene, G46630634C (rs4253778), is located in intron 7 [39].

Lopez-Leon et al. [40] conducted a systematic review and meta-analysis to assess whether there is an association between genotypes for this SNV and high sports performance in endurance. A total of 5 studies were analyzed [29,38,39,41,42] with the participation of 760 endurance athletes and 1792 non-athletes (control group). The athletes represented the following sports: rowing, marathon, biathlon, triathlon, cross-country skiing, swimming, speed skating and cycling. Three studies found a statistically significant association between the G allele and athletic endurance [29,38,42], while two studies reported no statistically significant association [39,41].

In the research by Ahmetov et al. [29] it has been found that the G allele is associated with increased oxidation of fatty acids in skeletal muscles and an increased proportion of slow twitch type I fibres. This type of muscle fibres uses oxygen more efficiently during aerobic exercises. Endurance athletes have relatively more type I slow fibres than fast skeletal muscle fibres, which allows sustained muscle contraction to be maintained over a long period of time. In addition, it has been shown that the homozygous GG genotype correlates with high values of the oxygen pulse [32].

Cieszczyk et al. [37] studied the association between alleles and genotypes G46630634C (rs4253778) of the *PPARA* gene and physical performance of endurance athletes and control group (non-athletes). The results showed that frequency of the homozygous GG genotype among athletes was 73.33% versus 54.70% among non-athletes (*p* = 0.04) and the G allele was 82.50% versus 70.17% (*p* = 0.01), respectively. The study confirmed a statistically significant higher frequency of G allele carriage in athletes performing aerobic exercise.

Thus, the G allele (SNV G46630634C) of the *PPARA* gene is associated with the status of an elite athlete training in sports with a predominantly aerobic orientation of physical activity.

### 3.5. CKM Gene

**Figure 6:**
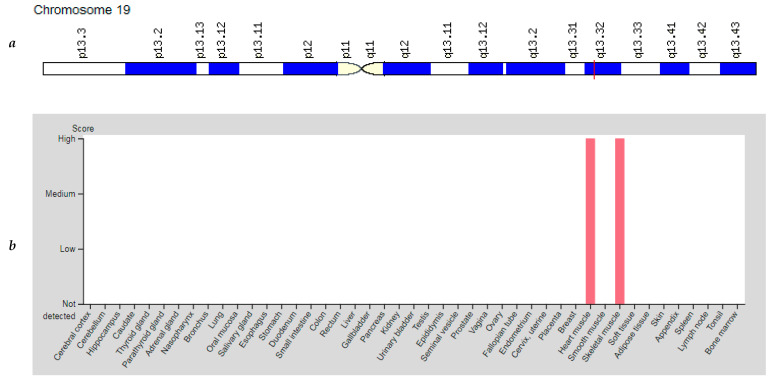
Location of the *CKM* gene (**a**) and tissue expression of the creatine kinase, muscle (**b**).

The *CKM* gene (Creatine Kinase, Muscle) is located on chromosome 19q13.32 (Figure 6a). The protein encoded by this gene is a cytoplasmic enzyme involved in energy homeostasis and is an important serum marker of myocardial infarction and muscular dystrophies [10].

The CKM is expressed at a high level in tissues with a high demand for energy, for example, in skeletal muscle and heart muscle (Figure 6b), which makes it possible to quickly restore ATP stores, which are the main source of energy in biochemical processes [43]. During exercises, adenosine diphosphate (ADP) accumulates in the contracting muscles and the creatine kinase mechanism of anaerobic ATP re-synthesis starts to work. The reaction is catalyzed by one of the key enzymes of energy supply for muscle activity—the muscle isoform of creatine phosphokinase (CKM). In the cell, CKM is a part of the M-band of striated muscles, which along with myomesin form M-bridges between myosin filaments. The protein is located on the surface of the myosin filament in the immediate proximity of the actomyosin ATPase and plays a part in the energy supply of the working myosin heads, supplying them with newly synthesized ATP in the process of muscle contraction. CKM localized on the surface of the endoplasmic reticulum affects the power of muscle contraction by regulating the flow of calcium ions during phases of tension and relaxation. Besides, along with the mitochondrial isoform of creatine phosphokinase CKM is involved in the transport of energy formed as a result of oxidative phosphorylation to muscle contractile proteins (creatine phosphate shuttle) [44]. Overall, most studies have supported the hypothesis that the *CKM* gene is a promising candidate gene associated with the development of aerobic capacity in elite athletes.

Over 260 SNVs have been described in *CKM* gene, where A45305950G (rs8111989) is the most frequently analysed [45]. There is evidence that this SNV is associated with better physical performance and contributes to differences in responses to MOC during endurance sports training [46]. The association between genotypes of SNV A45305950G of the *CKM* gene and the individual response of skeletal muscles of athletes to physical activity has been shown in several studies. It is believed that alleles and genotypes of this SNV can also be associated with different functional activities of *CKM* in myocytes. Chen et al. found that the power athletes had a significantly higher frequency of the G allele (OR, 1.14; 95% CI, 1.02–1.28, *p* = 0.03) and GG genotype (OR, 1.54; 95% CI, 1.24–1.91, *p* < 0.0001) compared to controls, but there was no significant difference for the endurance athletes (G allele, OR, 0.95, 95%CI, 0.85–1.06, *p* = 0.34; GG genotype, OR, 1.00, 95%CI, 0.78–1.27, *p* = 1.00). The results provide additional evidence to support the notion that human physical performance might be influenced by genetic profiles, especially in power sports [45].

He et al. [47] studied the nature of the distribution of genotypes in athletes and control group individuals in China. The authors found that genotypes of *CKM* gene A/G frequency in Uyghur general population were: AA—0.497; AG—0.392; GG—0.111. The result test by Hardy-Weinberg equilibrium and *x*^2^ = 2.72, *p* = 0.1, df = 2, indicated that the control group had representative. AA, AG and GG genotypes frequency of power-oriented athlete respectively was 0.442, 0.302 and 0.256. The frequency of GG genotype and G allele was higher than the control group, there were significant differences compared to the control (*p* < 0.05). A/G genotype frequency of Endurance-oriented athletes was 0.571, 0.400 and 0.029, there were no significant differences compared to the controls (*p* > 0. 05). A/G genotype frequency of Uyghur soccer athletes respectively was 0.472, 0.361 and 0.167. The frequency of G allele was higher than the Endurance-oriented athlete and lower than the power-oriented athletes, and no significant differences compared to the controls (*p* > 0.05).

Besides, the association of alleles and genotypes of this SNV with the aerobic capabilities of athletes, in particular MOC, was studied. MOC is a universal physiological indicator that reflects the level of physical fitness, especially aerobic capacity. Moreover, MOC has a high heritability (h2 = 0.59–0.87), and its level in the 18–26 age group reaches its maximum and remains relatively constant. It is from this perspective Gronek et al. [43] studied the association between SNV rs8111989 and MOC. The cohort was represented by 154 men and 85 women, including members of the Polish national team. The authors showed statistically significant differences in the frequency of the G allele among athletes and non-athletes. Specifically, the homozygous genotype AA was widespread among non-athlete women, in contrast to athletes who had a higher frequency of the AG and GG genotypes, especially among those involved in aerobic sports. The mean MOC was the lowest in homozygous carriers of the AA genotype and the highest in homozygous carriers of the GG genotype. The authors concluded that the rare (minor) G allele is a predictor of high MOC among athletes.

However, these results have not been confirmed for other ethnic groups or have been questioned. Thus, Rivera et al. [48] noted the highest MOC value among athletes with heterozygous AG genotype.

The muscle-specific creatine kinase enzyme (CK-MM) binds specifically to the M line of the myofibril subfragment, as well as to the outer membrane and vesicles of the sarcoplasmic reticulum of myocytes, which can affect the uptake of Ca^2+^ and the strength of skeletal muscle contraction. Type I (slow twitch) and type II (fast twitch) muscle fibres have been reported to differ in their CK-MM enzyme activity, with type I fibres showing at least half the activity of CK-MM. The skeletal muscles of athletes involved in endurance sports are characterized by a high proportion of type I fibres, as well as high levels of aerobic oxidative metabolism marker enzymes activity. Hence, lower CK-MM activity may be significant for endurance athletes [44].

Zhou et al. studied the association between the genotypes (AA, AG, and GG) of the rs8111989 SNV of the *CKM* gene and the individual response of running efficiency during endurance training. All indicators of running economy dropped markedly after training. Changes in oxygen consumption, changes in steady-state oxygen consumption in terms of average body weight and average lean body mass, as well as changes in ventilated volume in athletes with a heterozygous genotype AG were greater than in groups with a homozygous genotype GG. The authors concluded that the presence of the allele A may contribute to the individual response of running economics during endurance training [44].

During training some microdamages may appear on the myocyte membrane, through which the CK-MM enzyme enters the bloodstream. Its level in blood indicates exercise-induced muscle cell damage, myocardial infarction, or rhabdomyolysis. Heled et al. [49] studied the association between the carriage of SNV rs8111989 and the level of CR-MM after intense physical activity. The authors observed that the concentration of CK-MM in the blood was six times higher among athletes with a homozygous AA genotype than in other genotypes. Differences in blood levels of CK-MM in athletes after intense physical activity used in this study indicate that the physiological response was determined by the genotype. The authors suggested that the minor allele G may serve as a defence mechanism against exercise-induced muscle damage.

Chen et al. [45] published a meta-analysis devoted to the differences in the carriage of alleles (genotypes) of SNV rs8111989 of the *CKM* gene between athletes of strength sports, training for endurance, and control group individuals (non-athletes). Strength athletes had a higher frequency of carriage of the minor G allele (OR, 1.14; 95% CI, 1.02–1.28, *p* = 0.03) and the homozygous GG genotype (OR, 1.54; 95% CI, 1.24–1.91, *p* < 0.0001) compared to a control group. There were no statistically significant differences between strength and endurance athletes (allele G, OR, 0.95, 95% CI, 0.85–1.06, *p* = 0.34; genotype GG, OR, 1.00, 95% CI, 0.78–1.27, *p* = 1.00).

Fedotovskaia et al. [50] studied the distribution of frequency of alleles and genotypes of SNV A45305950G (rs8111989) of the *CKM* gene in athletes of various specializations (*n* = 384) and in the control group (*n* = 1116), as well as identifying the association of genotypes with aerobic performance. The frequency of the major allele A and homozygous AA genotype were significantly higher among endurance-oriented athletes than in the control group (allele A: 78.7% versus 65.4%; *p* < 0.0001; genotype AA: 59.7% versus 44.2%; *p* = 0.0003). On the other hand, the homozygous GG genotype was more common among weightlifters than in the control group (31.1% versus 13.4%; *p* = 0.0001). The homozygous AA genotype was associated with high MOC values (AA—58.98 (3.44) mL/kg/min, GA-56.99 (4.36) mL/kg/min, GG-52.87 (4.32) mL/kg/min, *p* = 0.0097).

The data obtained show that SNV A45305950G (rs8111989) of the *CKM* gene can predispose to individual differences in responses to sports training for endurance (major allele A) or strength (minor allele G).

Overall, most studies have supported the hypothesis that the *CKM* gene is a promising candidate gene associated with the development of aerobic capacity in elite athletes.

### 3.6. TFAM Gene

**Figure 7:**
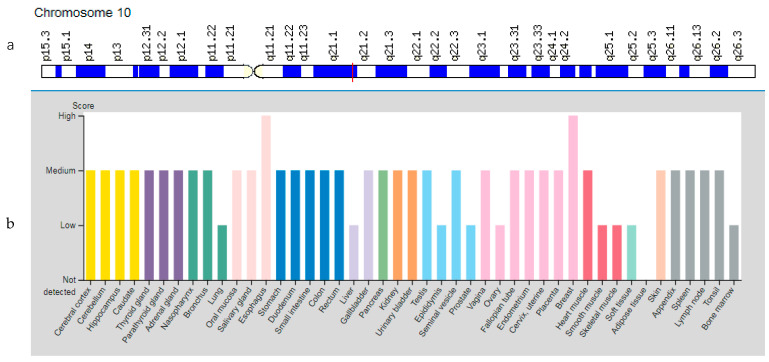
Location of the *TFAM* gene (**a**) and tissue expression of the transcription factor A, mitochondrial (**b**).

The *TFAM* gene (Transcription Factor A, Mitochondrial) is located on chromosome 10q21.1 (Figure 7a). The *TFAM* gene encodes a key protein responsible for the regulation of mitochondrial DNA replication and transcription and protects cells from oxidative stress [10]. As a result of training, the number of mitochondria in skeletal muscles increases, which increases their energy potential and reduces fatigability. At the same time, the *TFAM* transcription factor activity is the most important mechanism of mitochondrial biogenesis regulation in myocytes [51]. The protein is expressed in various organs and tissues, including skeletal muscles and heart muscles (Figure 7b).

Mitochondria play a leading part in the production of energy, which is necessary for physical exercises, especially aerobic exercises. The human mitochondrial genome encodes 13 proteins—components of enzymatic systems of oxidative phosphorylation, genes of two ribosomal and 22 transport RNAs. Maintaining the optimal amount of mitochondrial DNA (mtDNA) and the expression of its genes is a prerequisite for the aerobic energy supply of muscle activity [51].

Sports activities (aerobic or anaerobic) cause a change in molecular expression in skeletal muscles, which contributes to the adaptation of muscle tissue to the requirements of physical stress [52]. As a result, sport loads induce phenotypic changes in skeletal muscles, which include an increase in cross-sectional area, an increase in capillary density, a change in fibre type, and mitochondrial biogenesis, which leads to an increase in the density of muscle cell mitochondria [53,54]. Increasing mitochondrial density is a part of an adaptive process that allows the body under exercise stress to increase the amount of functional proteins involved in energy creation to cope with this specific stress later on. So, *TFAM* is the key mechanism for the activation of exercise-induced mitochondrial biogenesis [55].

Aerobic exercises lead to the increase in *TFAM* expression and mtDNA copy number. Ekstrand et al. [56] demonstrated in studies in mice that TFAM is a key regulator of mtDNA copy number. The introduced human *TFAM* gene was regulated in the same manner as the endogenous mouse *TFAM* gene, and expression of the human TFAM protein in mice did not lead to suppression of endogenous expression. Overexpression of TFAM protein among mice resulted in an overall increase in the mtDNA copy number, with the mtDNA copy number being directly proportional to the total TFAM protein level in mouse embryos.

The disruption the murine *TFAM* gene shows that heterozygous knockout mice have reduced mtDNA copy numbers and a deficiency of the respiratory chain in the heart. Larsson et al. [57].

The most significant SNV of the *TFAM* gene is G58385582C (rs1937), which results in the substitution of serine for threonine (Ser12Thr) in the encoded protein. Akhmetov et al. [51] studied the distribution of the frequency of alleles of a given SNV in non-athletes (control group). Aerobic capacity was determined by the value of the MOC and the indicator of the maximum load power on the ergometer. It was found that the frequency of the minor C (Thr) allele was significantly higher among endurance-oriented athletes than in the control group (14.0% vs. 9.1%; *p* < 0.0001). Besides that, the minor allele was associated with the qualification of the athlete (C allele frequency was among more qualified athletes). It was also found that the minor C allele is associated with high values of aerobic performance.

He et al. [58] studied the associations of SNVs (rs1937, rs2306604 and rs1049432) of *TFAM* gene with parameters of aerobic performance (MOC) and running economy before training and in response to 18- weeks of endurance training. The study involved 102 Chinese men (non-athletes). The authors did not find significant differences in the initial levels of MOC and running economy between genotypes or haplotypes of SNVs (rs1937, rs2306604, rs1049432) of the *TFAM* gene. Besides, no differences were found in the changes in studied parameters in response to the load. It was concluded that the three SNVs (rs1937, rs2306604, rs1049432) of *TFAM* genes do not predict endurance/learning ability, at least among Chinese men.

## 4. Discussion

To achieve elite status, an athlete must have certain physical qualities, which largely depend on genetic characteristics. In cyclic sports, varying from sprints, which last for a few seconds to marathons taking hours to run, the competitive result will be determined, first of all, by the availability of ATP for contractile activity of skeletal muscles. Therefore, the identification of genetic markers that determine the efficiency of the ATP re-synthesis pathways is one of the priority areas of physiology and sports genetics.

Each sport (speed-strength or endurance) will activate certain metabolic pathways (phosphogenic pathway, glycolytic pathway or mitochondrial respiration) to maintain the required rate of ATP re-synthesis. However, this thematic review demonstrates the polygenic nature of the regulation of energy metabolism in skeletal muscles (aerobic or anaerobic). Our review suggest that the likelihood of becoming an elite athlete in speed-strength sports or endurance sports depends on the alleles and genotypes various SNVs of candidate genes associated with the corresponding energy metabolism of skeletal muscles. The presence of certain SNVs of candidate genes by themselves cannot determine success or failure in sports, but can predispose to better sports performance with an adequate and individually selected training regime.

**Figure 8:**
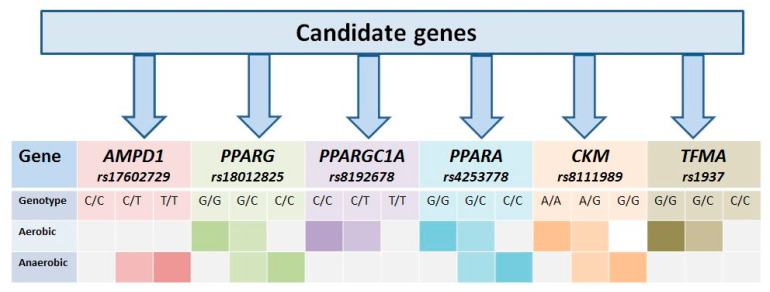
Personalized algorithm for determining the type of energy metabolism among athletes: The color saturation in squares corresponding to the variable genotypes of the candidate genes presented by SNVs demonstrates the best profile of energy metabolism of skeletal muscles and myocardium for anaerobic and aerobic sports.

So, our thematic review highlights the potential importance of candidate genes and their SNVs in sporting success in athletes of cyclic sport (Figure 8).

We considered three main conditions: the regulation metabolism of skeletal muscles:

- the regulation of aerobic metabolism of skeletal muscles: *PPARGC1A* (Gly/Gly) [7,33,34,36,59]; *PPARA* (G/G) [29,32,37,38,42,59]; *CKM* (A/A) [43,44,50]; *TFAM 12Thr)* [51,58];

- the regulation of anaerobic alactate (phosphogenic) metabolism of skeletal muscles: *AMPD1 (C/C)* [12,13,15]*; CKM (G/G)* [43,44,47,50];

- the regulation of anaerobic lactate (glycolytic) metabolism of skeletal muscles: *PPARG (Ala/Ala)* [16,22,23,25,26,27] *;* *PPARA (C/C)* [29,59].

## 5. Limitations

There are several limitations in our thematic research. We studied only Englishlanguage and Russian-language publications. Most of the publications cited for each gene always relate to one population (Polish, Russian, Chinese). The problem here is that the distribution of SNVs can be (are) population-specific. Further analysis of other populations is needed before making convincing statements about the prognostic role of the genes considered in the thematic review for sports genetics. Also, the limitations of translating these results into practice are the possibility of the influence of external factors, including nutrition, work and rest regime, intensity of the training process, gender and many other factors. It is not excluded that some SNVs may have an adaptive character for living in a particular environment. Finally, in recent years, the role of various SNVs of a large number of candidate genes in sports selection has been considered. It is important to analyze their mutual influence on sports performance for new thematic reviews in the future and to assess the possibility of translating the results of fundamental research into real clinical and sports practice.

## 6. Conclusions

Possible, identification of genetic biomarkers associated with the regulation of energy metabolism in skeletal muscles in athletes may help sports physicians and coaches develop personalized strategies for selecting children, teenagers and young adults for endurance, strength and speed sports. However, the multifactorial aspect of sport performances, including impact of genetics, epigenetics, environment (training, resting, nutrition, psycho-emotional status and etc.), is important for personalized strategies for selecting of athletes. This approach could improve sports performance and reduce the risk of sports injuries to the musculoskeletal system.

## Figures and Tables

**Table 1:** Genes responsible for myogenesis and skeletal muscle metabolism and diseases caused by their mutations (adapted from [9,10]).

Gene: MIM (Protein)	Location	Clinical Manifestations of Mutation: MIM	Inheritance
*AMPD1:* 102770 (Adenosine monophosphate deaminase 1 type)	1p13.2 (16 exons)	Myopathy Due to Myoadenylate Deaminase Deficiency: 615511	AD
*CKM:* 123310 (Creatine Kinase, Muscle)	19q13.32 (8 exons)	Myotonic Dystrophy: 160900	AD
*PPARA:* 170998 (Peroxisome Proliferator Activated Receptor Alpha)	22q13.31 (14 exons)	Fatty Liver Disease: 613282	Mu
*PPARG:* 601487 (Peroxisome proliferator activated receptor gamma)	3p25.2 (14 exons)	Lipodystrophy, Obesity, Diabetes Mellitus Type 2 Familial Partial Lipodystrophy Type 3: 604367	Mu AD
*PPARGC1A:* 604517 (Peroxisome Proliferator-Activated Receptor Gamma Coactivator 1 Alpha)	4p15.2 (24 exons)	Amyotrophic Lateral Sclerosis Type 1: 105400 Huntington Disease: 604802	AD/AR AR
*TFAM:* 600438 (Transcription Factor A, Mitochondrial)	10q21.1 (8 exons)	Mitochondrial DNA Depletion Syndrome Type 15: 617156	AR

**Table 2:** Candidate genes and their encoded proteins and enzymes involved in the regulation of energy metabolism in skeletal muscles (adapted from [10]).

Gene	Localization, Chromosome	Protein/ Enzyme	Effects on Energy Metabolism of Skeletal Muscle
*AMPD1*	1p13.2	Adenosine monophosphate deaminase 1 type (AMPD1)	AMPD1 catalyzes the deamination of adenosine monophosphate and inosine monophosphate in skeletal muscle and plays an important role in the purine nucleotide cycle.
*PPARG*	3p25.2	Peroxisome proliferator activated receptor gamma (PPARG)	PPARG controls the peroxisome beta-oxidation pathway of fatty acids and is a key regulator of adipocyte differentiation and glucose homeostasis.
*PPARGC1A*	4p15.2	Peroxisome Proliferator-Activated Receptor Gamma Coactivator 1 Alpha (PPARGC1A)	PPARGC1A regulates genes involved in energy metabolism, provides a direct link between external physiological stimuli and regulation of mitochondrial biogenesis, and is the main factor regulating the determination of muscle fiber type.
*PPARA*	22q13.31	Peroxisome Proliferator Activated Receptor Alpha (PPARA)	PPARA is involved in the regulation of energy metabolism, regulates the expression of genes encoding several key muscle enzymes involved in fatty acid oxidation.
*CKM*	19q13.32	Creatine Kinase, Muscle (CKM)	CKM catalyzes the transfer of phosphate between ATP and various phosphogenic groups such as creatine phosphate; CKM isozymes play a central role in energy transduction in tissues with high energy requirements such as skeletal muscle, heart, brain.
*TFAM*	10q21.1	Transcription Factor A, Mitochondrial (TFAM)	TFAM is responsible for regulating mitochondrial DNA replication and transcription and also protects cells from oxidative stress.

**Table 3:** Genes responsible for energy metabolism and their expression in skeletal muscle, myocardium and lungs (adapted from [11]).

Gene	Expression Level in Skeletal Muscles (RPKM)	Expression Level in Myocardium (RPKM)	Expression Level in Lung (RPKM)
*AMPD1*	225.7	0.023	0.363
*PPARG*	2.097	4.716	19.45
*PPARGC1A*	11.02	9.406	3.292
*PPARA*	12.34	7.889	5.624
*CKM*	25890.0	2987	4.667
*TFAM*	6.0	4.754	9.471
